# UTILLdb, a *Pisum sativum in silico *forward and reverse genetics tool

**DOI:** 10.1186/gb-2008-9-2-r43

**Published:** 2008-02-26

**Authors:** Marion Dalmais, Julien Schmidt, Christine Le Signor, Francoise Moussy, Judith Burstin, Vincent Savois, Gregoire Aubert, Veronique Brunaud, Yannick de Oliveira, Cecile Guichard, Richard Thompson, Abdelhafid Bendahmane

**Affiliations:** 1 Unité de Recherche en Génomique Végétale, UMR INRA-CNRS, Rue Gaston Crémieux, 91057 Evry Cedex, France; 2 INRA, Unite Mixte de Recherche en Génétique et Ecophysiologie des Légumineuses (INRA-ENESAD), Domaine d'Epoisses, 21110 Bretenières, France

## Abstract

The systematic characterization of gene functions in species recalcitrant to *Agrobacterium*-based transformation, like *Pisum sativum*, remains a challenge. To develop a high throughput forward and reverse genetics tool in pea, we have constructed a reference ethylmethane sulfonate mutant population and developed a database, UTILLdb, that contains phenotypic as well as sequence information on mutant genes. UTILLdb can be searched online for TILLING alleles, through the BLAST tool, or for phenotypic information about mutants by keywords.

## Abstract 2

UTILLdb is a database of phenotypic and sequence information on mutant genes from a reference Pisum sativum EMS-mutant population.

## Background

Mutational approaches have been widely exploited in breeding and basic research. In the genomic era, the completion of the sequencing of several plant genomes has enabled the development of reverse genetics strategies, where one first identifies a target gene based on the functional annotation of its sequence, and then proceeds with the phenotypic characterization of mutant alleles. Several mutagenesis techniques are dedicated to this approach, notably RNA interference suppression [1,2] and insertional mutagenesis by transposon tagging [3,4] or *Agrobacterium *T-DNA insertion [5]. These methods, however, are still mainly based on *Agrobacterium *T-DNA vectors and, thus, rely on the ability of a given plant species to be transformed. On the other hand, chemical mutagenesis based on an alkylating agents like ethylmethane sulfonate (EMS) [6] provides an easy and cost-effective way to saturate a genome with mutations. TILLING (targeting induced local lesions in genomes) uses EMS mutagenesis coupled with gene-specific detection of single-nucleotide mutations [7-9]. This reverse genetic strategy encompasses all types of organisms [10-14] and can be automated in a high throughput mode, which is an absolute necessity to match the speed of candidate gene discovery.

The success of the TILLING approach relies on the construction of high quality mutant libraries. Ideally, the mutant population is phenotyped so that *in silico *analysis of the mutant lines can be carried out. To date, phenotypic databases can be found for tomato [15], rice [16], *Lotus japonicus *[13] and *Arabidopsis *[17], and a searchable collection of phenotypic mutants is available for *Zea mays *[18], *Pisum sativum *[19] and *Arabidopsis thaliana *[20].

Pea (*P. sativum*) belongs to the Leguminoseae family, which provides excellent dietary components with health-promoting benefits and offers the important ecological advantage of contributing to the development of low input farming systems by fixing atmospheric nitrogen and further minimizing the need for external inputs when used as a break crop. Since Gregor Mendel's groundbreaking work on the theories of heredity, pea has been extensively used for basic research, in particular in the fields of seed biology and plant architecture. In many studied examples, legume genes were shown to have novel functions compared to those described for related *Arabidopsis *genes. Detailed characterization of these legume genes will help our understanding of cross-species gene function [21]. However, functional gene validation by transformation is impractical due to the difficulty of transforming pea using *Agrobacterium*. This situation renders pea an ideal candidate for TILLING. Although several pea EMS mutant populations already exist, they are unsuitable for a genomic approach as they have not been prepared or maintained under rigorously controlled conditions and suffer from cross-contamination. Hence, there is a need for a high-quality *P. sativum *genetic mutant reference collection, which could be used for both forward and reverse genetics studies. Within the frame of the European Grain Legumes Integrated Project [22], we have developed such a population by mutagenizing *P. sativum *cultivar Caméor with EMS, and establishing an associated TILLING platform and phenotype database, UTILLdb.

## Results

### Production of Caméor mutant population

Caméor is an early-flowering garden pea cultivar that completes its reproductive cycle within four months, permitting three successive generations a year under greenhouse conditions. Although pea is predominantly self-fertilizing, some residual cross-pollination can occur. In order to avoid contamination, 100 Caméor plants, derived from single seeds, were analyzed for genetic uniformity using a set of 16 short sequence repeat markers distributed over every arm of the seven predicted pea chromosomes [23] and left to set seeds in insect-proof greenhouses. In total, 10,000 Caméor seeds were produced and used to create the mutant population.

**Figure 1:**
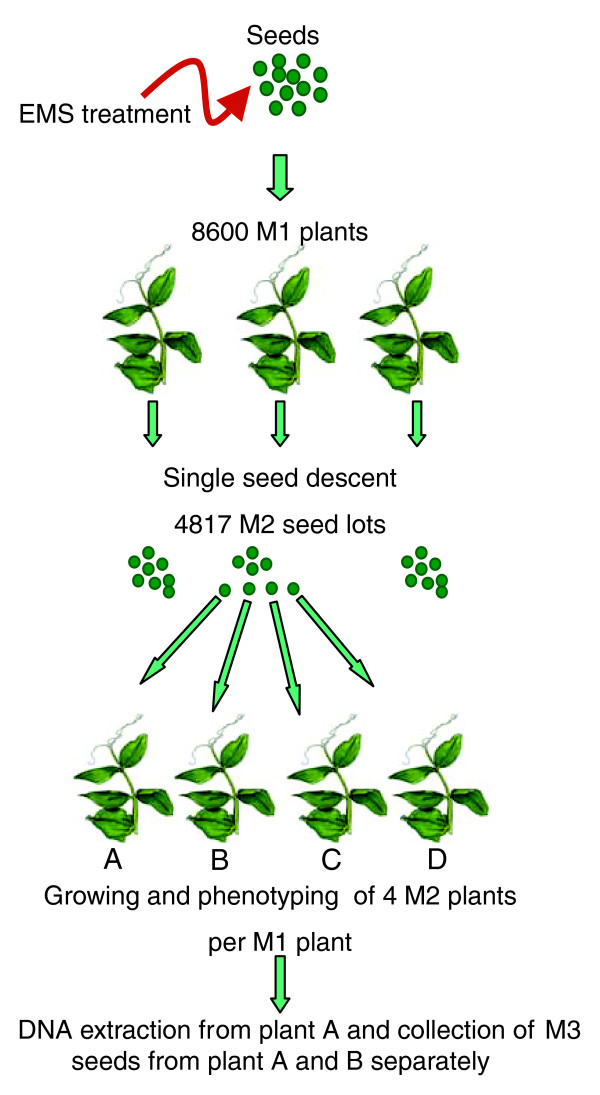
Establishment of pea EMS mutant library. Caméor seeds were EMS mutagenized. Out of 8,600 M1 plants self-fertilized in an insect-proof glasshouse, 4,817 produced more than 5 M2 seeds each. Four M2 seeds, referred to as A-D, per M1 parent were grown to maturity and scored for phenotypes. DNA was extracted from the plants referred to as A, which were left to set M3 seeds. As a backup, M3 seeds were harvested from the sister B plants. The collected M3 seeds were sent to the Grain Legumes Biological Resource Center for distribution, maintenance of the lines and long-term storage of the mutant library.

In order to balance maximum mutation density with acceptable plant survival rate, we first conducted a 'kill-curve' analysis on batches of 100 seeds, using a range of doses from 8 to 57 mM EMS. Most treated first generation mutant (M1) plants exhibited retarded growth at an early seedling stage, but all of them recovered. Thirty plants from each treatment were then grown until maturity and assessed for fertility and seed production. A high loss of fertility was observed at the highest doses, with less than 30% of plants fertile at doses higher than 32 mM EMS. The highest EMS doses allowing 50% of plants to set seeds, 16 mM and 24 mM, were retained and tested on large batches of seeds (Table 1). Little difference was observed between these two doses with a tendency toward higher seed production with 16 mM EMS, so a final dose of 20 mM EMS was used for population production. The mean number of seeds per pod was also slightly higher for the plants treated with 16 mM than for those treated with 24 mM EMS. The high rate of arrested embryos in pods of M1 plants treated with EMS doses of 16-24 mM attested to its good mutagenesis efficacy. Out of 8,600 M1 plants, more than 4,817 lines that had produced more than 5 M2 seeds each were individually harvested. To produce M3 seeds, four M2 seeds per M1 plant were sown in two-liter pots and M3 seeds were harvested from two sister plants, referred to as A and B. Leaf material was harvested from the healthiest looking plant, referred to as A (Figure 1). Seed stocks were sent to the Grain Legumes stock center in Dijon for multiplication, distribution and long-term storage of the lines.

**Table 1:** Effect of EMS

Dose of EMS	0 mM	16 mM	20 mM	24 mM
Total M1 seeds sown	100	1000	4000	3600
Percentage of M1 plants setting seeds	100%	61%	63%	58%
Percentage of M1 plants yielding more than 5 seeds	100%	56%	52%	39%
Percentage of arrested embryos in pods of M1 plants	3%	45%	49%	52%
Mean number of seeds per pod (± SD)	4.83 ± 0.91	2.00 ± 0.86	0.91 ± 1.30	0.79 ± 1.93

Effect of the concentration of EMS on M2 seed setting, on the frequency of arrested embryos (data are expressed as the percentage of total seeds analyzed) and on the mean number of seeds per pod in the M1 generation (200 pods analyzed per dose). SD, standard deviation.

### Phenotyping of the Caméor mutant population

As we intended to create a reference mutant collection that could be used for forward and reverse genetics, we carried out a systematic phenotyping of the mutant population. Our phenotype scoring was based on visual characterization of four plants per M2 family at key developmental stages, from germination until fruit maturation. To facilitate the phenotype scoring we defined a phenotype ontology adapted to pea. This phenotyping tool does not cover all phenotypic alterations (for example, no root evaluation was carried out) and was constructed for high-throughput scoring of many mutant lines in a relatively short growing season. The vocabulary used to describe the mutant plants was organized in a hierarchical tree and is composed of 107 subcategories of phenotypes clustered at different levels. The complete list of the vocabulary used is shown in Additional data file 1 and the number of lines found in each major phenotype category is shown in Table 2.

**Table 2:** Number of M2 families affected in the major categories and sub-categories of phenotypes

Major category		Subcategory	No. of families
1	Cotyledon	Color	172
		Shape	32
2	Plantlet architecture	Architecture	7
3	Plant architecture	Architecture	316
		Branching type	205
4	Leaf	Color	610
		Shape and arrangements	387
		Appearance	253
		Size	81
5	Stipule	Size/color/shape	77
6	Petiole	Petiole	6
7	Stem	Stem size	1,447
		Shape	36
8	Flower	Flower morphology	24
		Flowering time	4
		Reproductive organs	12
9	Seed	Seed color	2
		Shape	4
		Size	66

**Figure 2:**
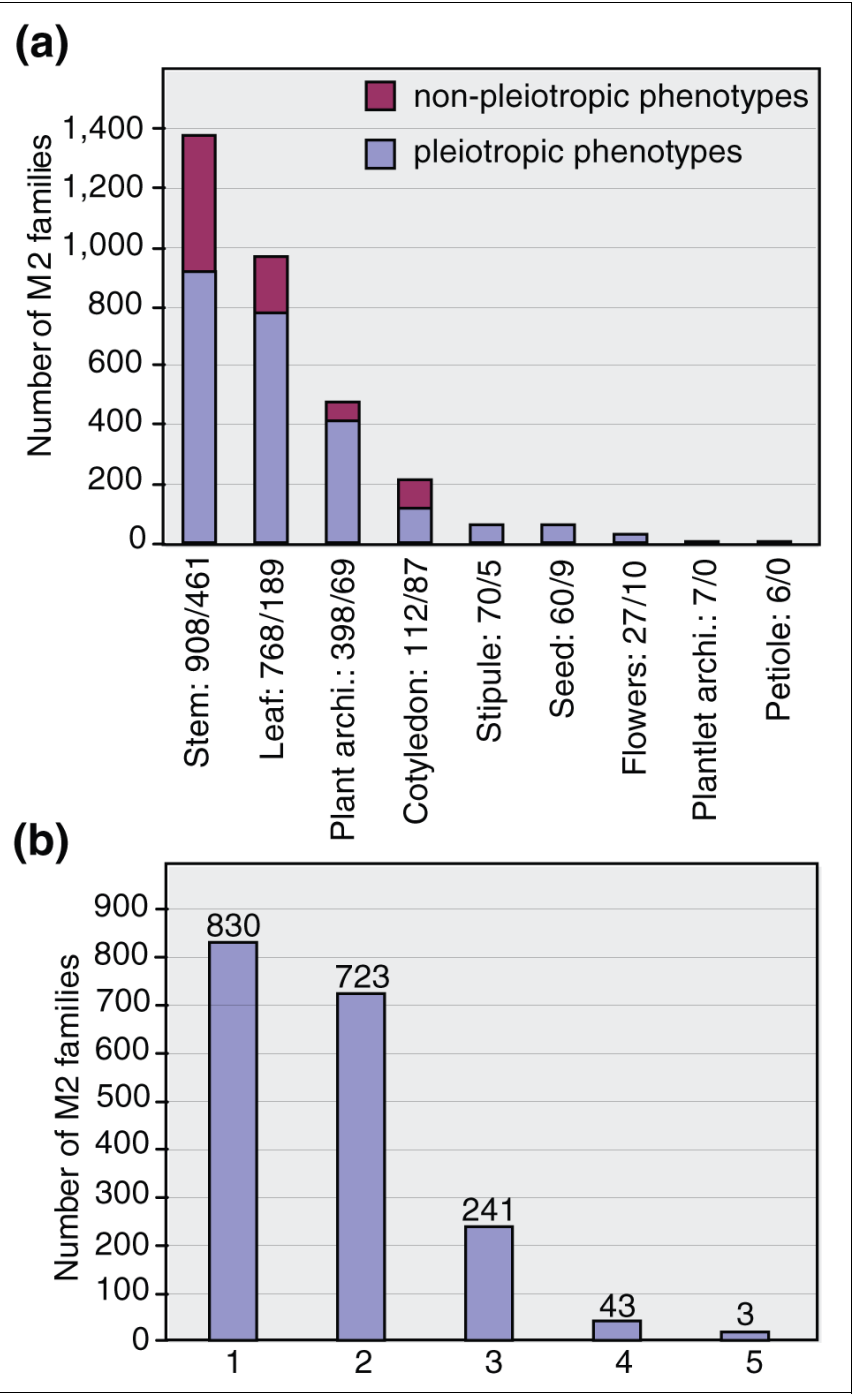
Distribution of phenotypic characteristics of the mutant population and rate of pleiotropy. **(a) **Number of M2 families in each phenotypic group. The x-axis indicates the nine major phenotypic categories, listed in Table 2, and the y-axis indicates the total number of M2 families. Each bar represents the number of mutants in the corresponding category. The blue bar represents the quantity of pleiotropic mutants (having more than one phenotype), given by the first number in the category label. The red bar represents the non-pleiotropic mutants and is given by the second number in the category label. **(b) **Total number of M2 families (y-axis) sharing 1-5 major phenotypic categories (x-axis). The bar for one phenotypic category indicates how many mutants are categorized in only one phenotypic group (non-pleiotropic mutants), and the bars for the 2-5 phenotypic categories represent the number of mutants that share two to five phenotypes, respectively. In each case, the total number of mutants is indicated on the top of the bar.

**Figure 3:**
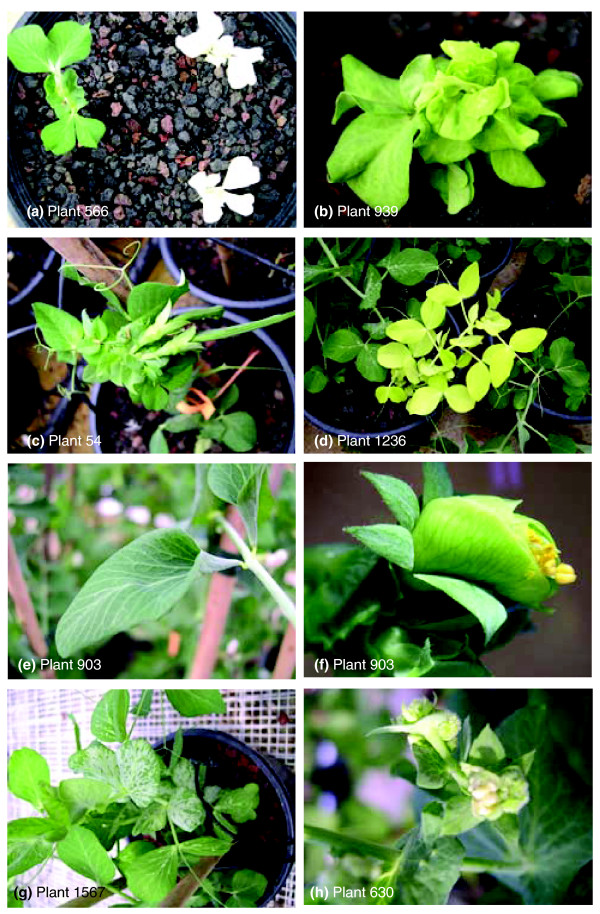
Examples of mutant phenotypes representing the nine major phenotypic groups. **(a) **Plant 566: cotyledon color, albino. **(b) **Plant 939: plantlet architecture, bushy; plant architecture, hyper compact; leaf color, pale green; stem size, extreme dwarf. **(c) **Plant 54: plant architecture, determinate growth. **(d) **Plant 1,236: plant architecture, basal branching; leaf color, pale green, yellow; leaf size, medium; stem size, dwarf. **(e, f) **Plant 903: leaf, cone shaped at leaf base; flowers, sterile flowers. **(g) **Plant 1,567: leaf, distorted; stipule, silver-argentous. **(h) **Plant 630: flowers, cauliflower type inflorescence; flowers, abnormal all; stem, dwarf; leaf, upcurling.

Out of the 4,817 M2 families, 1,840 showed a visible phenotype, which represents 38% of the lines. Among the lines that showed a visible phenotype, 45% were scored for a single phenotype and 55% displayed multiple phenotypes, that is, they fall into more than one major phenotype category (Figure 2a). This rate of pleiotropy is an underestimation as the phenotypic characterization is based on high-throughput visual observation of only four mutant lines per M2 family. Detailed morphological and biochemical characterization of higher numbers of plants per M2 family would result in more phenotypic effects per mutant and, thus, a higher rate of pleiotropy. The most commonly observed phenotypes are related to stem size, leaf and plant architecture, followed by those related to cotyledons, stipules and seeds, with the least abundant phenotypes being related to flowers, plantlet architecture and petiole morphology (Figure 2b). Examples of phenotypes corresponding to the primary categories described are shown in Figure 3.

### Caméor TILLING platform

To set up the pea TILLING platform, DNA samples were prepared from 4,704 M2 plants, each representing an independent family and organized in pools of 8 M2 families. One key factor in TILLING is the availability of the annotated genomic sequence of the gene to be tilled. Even though the pea genome has not yet been sequenced, acquisition of the genomic sequences of target genes is facilitated by the high degree of synteny between pea and the model plant *Medicago truncatula*, which is being sequenced [24]. The CODDLE program (Codons Optimized to Discover Deleterious Lesions [25,26]) combined with the PRIMER3 tool [27] are used to define the best amplicon for TILLING. PCR products used for TILLING have a maximum size of about 1,500 bp and, therefore, longer genes are divided into several amplicons. To reduce variation in the quality and the quantity of the PCR amplification product due to the pea genome complexity and low amount of genomic DNA used in PCR, nested PCR is performed. Mutations are detected in the amplified targets using the mismatch-specific endonuclease ENDO1, as described previously [28]. Individual mutant lines are identified following a pool deconvolution step, and then the mutated base is identified by sequencing.

**Figure 4:**
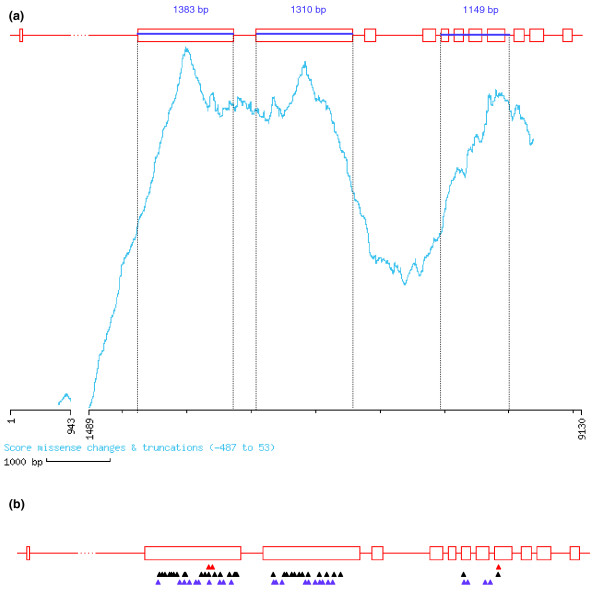
Comparison between predicted and obtained mutations. **(a) **Output of the CODDLE program using as an example the *PsMetI *genomic sequence. Exons are represented by white boxes and introns by red lines. The CODDLE program was used to identify those regions of the gene in which G:C to A:T transitions are most likely to result in deleterious effects on the encoded protein (represented by the probability curve traced in tourquoise). The CODDLE algorithm is based on an evaluation of protein sequence conservation from comparison of database accessions of homologous proteins. For *PsMetI*, three fragments were chosen based on these CODDLE results (blue lines). External and internal primers were designed to amplify each region by nested PCR. **(b) **Graphic representation of mutations identified in the three regions of the gene *PsMetI*. This drawing was made using the PARSESNP program [43], which maps the mutation on a gene model to illustrate the distribution of mutations. Purple triangles represent silent mutations and black and red triangles represent missense and truncation mutations, respectively.

**Figure 5:**
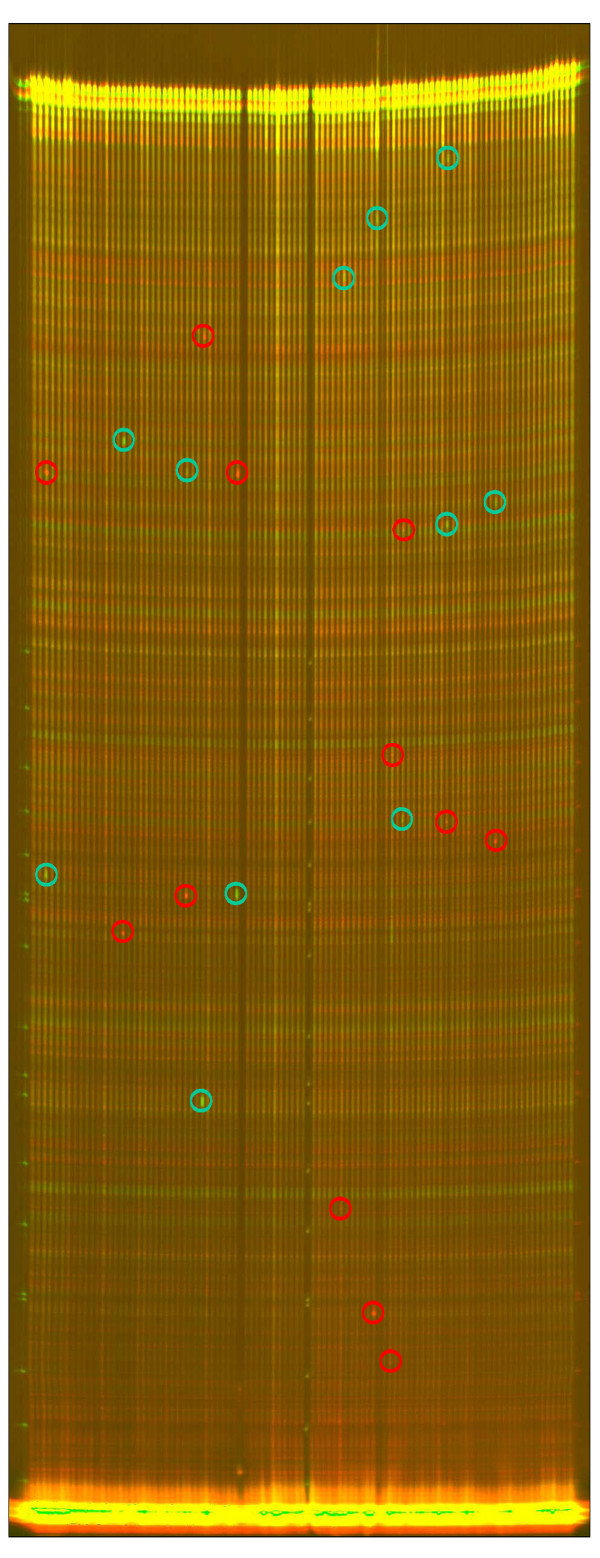
TILLING screen. Example of a *PsMetI *TILLING screen on eightfold pooled pea DNA. The image of the cleavage reaction is collected from both channels (dyes IRD700 and IRD800). The sizes of the cleavage products (circled) from the two dye-labeled DNA strands (red or green) add up to the size of the full-length PCR product (top of the gel). PCR artifacts are distinguishable from true mutants by yellow points (red and green added) as they appear at the same size in both channels. The size of the cleavage product (the sizing ladder can be seen at the left and middle of the image) indicates approximately where the single nucleotide polymorphism is located in the fragment.

A primary objective in a mutagenesis project is to generate a saturated resource where every locus is mutated and represented by multiple alleles. To evaluate the existence of multiple alleles per locus, we screened for mutations in the pea Methyl transferase 1 gene (*PsMet1*) [29]. Three amplicons of 1,383, 1,310 and 1,149 bp were tilled (Figure 4) and 96 mutants were identified (Figure 5). Sequence analysis of the mutations showed that 6 were intronic, 37 silent, 50 missense and 3 nonsense mutations (Figure 4b). Although characterization of *PsMet1 *mutants is beyond the scope of this article, we found that retrieval of the mutant alleles from the A plant M3 seed stocks was successful, without the need to use backup M3 seed stocks collected from the sister B plants (Figure 1). The exonic mutants were mostly present as heterozygotes (79 out of 90 mutations), but 11 lines were homozygous for the mutations. As expected with EMS mutagenesis, these mutations were distributed relatively evenly within the screened amplicons (Figure 4b).

To further evaluate the quality of the mutant population, we extended the TILLING screen to another 19 genes and identified 371 point mutations in those genes (Table 3). As expected for EMS, all the mutations were G:C to A:T transitions [6,30]. Induced mutations discovered in exons consisted of 66.75% missense, 28.51% silent and 4.74% stop mutations (Table 4). Although the number of observed missense mutations was bigger than the amount predicted by CODDLE (63.80%), we recovered stop mutations in a slightly lower proportion than predicted (6.90%). As many tilled amplicons harbor intronic segments, some recovered mutations were intronic. Although some of these could potentially affect the efficiency of mRNA splicing, such an impact is unpredictable. Thus, intronic mutants were not characterized further. In contrast, the large number of non-synonymous mutations recovered is of interest as they may lead to gain- or loss-of-function phenotypes. Such mutations will also permit dissection of the function of the protein with respect to its sub-domain structure.

**Table 3:** Tilled genes and mutation density in Caméor mutant population

Tilled genes	Amplicon size (bp)	% of GC in exons	Identified mutants	Screened M2 families	Mutation frequency
Ps CONSTANS-like a (*PsCOLa*)	1,012	46.30%	11	1,536	1/141 Kb
*LectineA*	971	40.80%	13	1,536	1/115 Kb
Sucrose transporter (*SUT1*)	1,014	52.40%	12	1,536	1/130 Kb
Cell wall invertase (*cwINV*)	1,612	41.50%	12	1,536	1/206 Kb
Serine-threonine proteine kinase (*Sym29*)	2,457	44.00%	8	768	1/236 Kb
Phosphoenolpyruvate carboxylase (*PepC*)	1,009	44.40%	25	3,072	1/124 Kb
Lec1-like (*L*1*L*)	870	39.10%	21	4,608	1/191 Kb
DOF transcription factor 2 (*PsDOF2*)	1,200	36.60%	9	3,072	1/410 Kb
Trypsine inhibitor (*TI1*)	712	34.20%	13	3,840	1/210 Kb
Pea albumine (*PA2*)	746	38.50%	9	3,072	1/255 Kb
Anther specific protein (*End1*)	851	40.50%	31	3,072	1/84 Kb
MADS box gene (*PM10*)	1,302	34.60%	20	4,608	1/300 Kb
MADS box gene (*PM2*)	1,390	31.30%	28	4,608	1/229 Kb
Tendril-less transcription factor (*TL*)	1,104	34.00%	28	3,072	1/121 Kb
Eukaryotic translation initiation factor (*eiF4e*)	1,383	36.90%	36	4,608	1/177 Kb
Eukaryotic translation initiation factor (*eIF(iso)4e*)	772	36.70%	10	4,608	1/356 Kb
Methyl transferase 1 (*Met1*)	3,842	40.20%	96	4,704	1/188 Kb
Retinoblastoma related (*RBR*)	2,959	40.80%	72	4,608	1/112 Kb
Late embryogenesis abundant protein (*PsLEAM*)	952	44.00%	17	4,608	1/258 Kb
Heat shock protein 22 (*HSP22*)	622	45.66%	18	4,608	1/159 Kb
Total/mean	26,780	40.12%	467	-	1/200 Kb

Part or all of the Caméor mutant population was screened for mutations in the genes listed. The size of the screened amplicon, the number of mutants identified and the mutation frequency for each amplicon are indicated. The average mutation frequency was estimated to one mutation per 200 kb and is calculated as in Greene *et al*. [6], except that we have summed the sizes of all the amplicons and we divided by the total number of identified mutants.

**Table 4:** Mutation types

	All	Silent	Missense	Truncation
Percent expected (CODDLE)	100	29.30	63.80	6.90
Percent observed	100	28.51	66.75	4.74
Percent heterozygous	86.60	27.7	56.4	2.5
Percent homozygous	13.34	4.78	8.06	0.5

Comparison of expected and observed types of mutations in tilled exonic regions; distribution between heterozygous and homozygous states in the mutant lines. The percentage of expected mutations was calculated by adding the results of CODDLE analysis, on the amplified regions only, for each gene.

We calculated the mutation frequency in the 20 targeted genes (Table 3) according to Greene *et al*. [6]: mutation frequency equals the size of the amplicon multiplied by the total number of samples screened divided by the total number of identified mutants. We estimated the average mutation rate to be one mutation every 200 kb. This mutation density is 1.5 times higher than the rate of one mutation per 300 kb reported for *Arabidopsis*, the best characterized TILLING mutant population to date [6]. Therefore, the 16-24 mM dose of EMS used to create the pea mutant population appears to be an adequate dose for TILLING. On average, we identified 34 alleles per tilled gene (after normalization to TILLING of the entire population). Considering that about half of missense mutations should have a deleterious effect on a typical protein [31], 25 alleles per tilled kilobase would be sufficient for phenotypic analyses.

### Setup of the UTILLdb database

We scored 4,817 lines in the mutant population for phenotypic alterations using 107 subcategories of phenotypes. In TILLING screens we searched for mutations in 20 genes and identified 467 alleles. In order to manage and integrate the expanding data from both the phenotype recordings and TILLING target genes, we implemented the database UTILLdb. UTILLdb was developed according to a relational database system, interconnecting four main modules: lines, phenotype categories, sequences and mutations. Two main types of data are accessible, the morphological phenotypes of mutants and the sequences of tilled genes and corresponding alleles, when available. UTILLdb may be searched using a sequence, through a BLAST tool [32] or for a phenotypic feature using a keyword search. The outcome of the search is shown as a table of results that displays the phenotype of each line, with associated pictures and mutated sequence if it exists. Thus, the user could ask whether lines that share mutations in a specific gene share the same phenotypes and vice versa. As we expect the phenotypic characterization of the TILLING mutants to become more detailed as they are analyzed by UTILLdb users, UTILLdb was designed so that the passport data of the mutant lines can be extended or modified as needed. UTILLdb is publicly accessible through a web interface [33]. A link is implemented to facilitate seed ordering. UTILLdb serves also as an entry point for users wishing to have their favorite gene tilled on the Caméor TILLING platform. Results from those screens as well as the phenotype of the mutants identified will be implemented in UTILLdb.

## Discussion

### Mutant population for forward and reverse genetics

EMS-mutagenized populations have been created for different crops with, in many cases, multiple populations per crop. Information on the quality of the mutagenesis and the production and maintenance of the seed stocks are, however, often unavailable. We have constructed a reference EMS mutant population from *P. sativum *cultivar Caméor under controlled conditions and developed a database, UTILLdb, which presents phenotypic data based on visual characterization of M2 plants from young seedling to fruit maturation stages. A hierarchical categorization of mutant phenotypes was used to describe the mutant plants. To facilitate the phenotype description, digital images were also recorded. We did not implement the previously published plant phenotype ontology [34,35], a hierarchical description intended to develop a vocabulary that describes anatomy, morphology, and growth and developmental stages of a flowering plant, for the main reason that the plant phenotype ontology vocabulary is not yet adapted to describe mutant morphological traits in a crop like pea. Instead, the vocabulary used to describe the pea mutant plants was inspired from previous investigations of mutant collections (tomato [15], lotus [13], barley [36]) and adapted to pea.

Tto exploit the mutant population using reverse genetics, genomic DNA was prepared from the mutant lines via high-throughput automated protocols, and organized in pools for bulked screening. Individuals with mutations in the gene of interest were isolated by systematic pool deconvolution. Genes and mutations were integrated in UTILLdb through a web interface, which allows for global analysis of the TILLING mutants in the collection. This database also serves as a portal for users to request materials or TILLING experiments.

### Saturation of the mutation screen

EMS mutagenesis causes primarily G:C to A:T transitions [30]. In the TILLING screen for mutations in *PsMet1*, we identified 90 independent exonic mutations in a sequence that contains 1,434 cytosines and guanines and this in a mutant population of 4,704 M2 families. Based on this we estimated the average frequency of mutations to be 1.33 × 10^5 ^(90/4,704 × 1,434). Given a genome size of 5,000 Mb and a 43.23% G:C content in the coding sequence of the pea genome [37], there are 2.2 × 10^9 ^bp susceptible to EMS mutagenesis. Assuming that all G:C base pairs are equally sensitive to EMS, we would expect approximately 2.93 × 10^4 ^mutations in each EMS-mutagenized M_1 _plant ((1.33 × 10^-5^) × (2.2 × 10^9^)). We used the binomial distribution, *P *= 1 - (1 - *F*)^*N*^, to calculate the probability of finding a mutation in a given G:C base pair in our mutant population. In this formula, *P *is the probability of finding the mutation, *F *is the mutation frequency per base pair (1.33 × 10^-5^), and *N *is the number of M1 mutant lines (4,704). Using this formula we estimated the probability of finding one mutation in any given G:C base pair in the genome as 0.06%. Increasing the size of the mutant population to 50,000 M2 plants raises the probability of finding one mutation in any given G:C base pair in the genome to 52%. This number is relatively small and could be managed by our platform. In fact, 50,000 independent lines represent 65 DNA pool plates (96-wells) or only 16 plates (384-wells). This purely theoretical example shows that EMS mutagenesis coupled with TILLING is a very powerful tool for creating genetic diversity, especially if one considers that routine transformation of *P. sativum *has not yet been achieved and, hence, insertional mutagenesis is not an option.

### Analysis of mutants identified through TILLING

The calculated overall mutation rate of one mutation every 200 kb found in our population is intermediate between the rate of one mutation per 300 kb reported for *Arabidopsis *[6] or C*enorhabditis elegans *(1/293 kb) [38] and rice [39], and 2.5-fold higher than the rate of two mutations per megabase for TILLING in maize [40]. A much more saturated mutation density has been observed in tetraploid wheat (1/40 kb), hexaploid wheat (1/24 kb) [41] or *Brassica napus *(1/10 kb; unpublished data); however, such species are able to withstand much higher doses of EMS without obvious impact on survival or fertility rates, due to multiple gene redundancies in their polyploid genomes.

In the TILLING screen, we recovered from 8 (*Sym29*) to 96 mutants (*PsMetI*) per tilled gene. Some genes (*End1*, *TL*) are obviously much more mutated than others (*DOF2*, *eIF(iso)4e*), despite the similarity of their GC content (36.6% for *DOF2*, 34% for *TL*). Of course, the propensity of a gene to withstand mutations without the resulting protein causing deleterious effects on the plant plays a major role and gametophytic lethal mutations will never be found in the population. However, we could see that some primer pairs used for screening gave a higher background noise than others, which affects the discrimination between true mutants and false positives on the polyacrylamide gel image, and reduces the number of mutants recovered. Nevertheless, our average score of 34 mutant alleles identified per tilled gene is higher than the 10 mutations per gene of *Arabidopsis *[6] or rice [39].

Screening for mutations in *PsMet1 *resulted in 96 alleles, of which 50 were missense and 3 non-sense mutations; in this case, the large number of mutations recovered is, at first sight, impressive, but the large gene size and targeted region (3,842 bp), together with the fact that we tilled the entire population (4,704 lines), accounts for this result. On the other hand, this example illustrates the strength of TILLING when it comes to finding a specific point mutation.

Because of the high number of alleles we routinely identify, the possible impact of missense mutations on the function of a protein is assessed before systematic phenotyping of the mutant plants, using two different programs: SIFT (Sorting Intolerant From Tolerant) [42], which uses PSI-BLAST alignments, and PARSESNP (Project Aligned Related Sequences and Evaluate SNPs) [43], which provides a position-specific scoring matrix based on alignment blocks (Figure 4d). In the case of *PsMet1*, 13 out of the 50 missense mutations (23%) were predicted to have a major impact on the function of the protein. Thus, the corresponding 13 mutant lines are characterized first.

In *Arabidopsis*, the *MetI *gene controls maintenance of CpG methylation [29]. It was previously shown that point mutations in *AtMetI *can lead to genome hypomethylation [29,44] with a variable impact on plant development, ranging from a late-flowering phenotype to reduced embryo viability. *P. sativum *has a genome mainly composed of non-coding repeated sequences [45], which are typically subjected to chromatin-mediated epigenetic suppression of transcription [46], in which an elevated rate of DNA methylation plays a major role. We intend to investigate the stability of those regions in a hypomethylated context, that is, in *PsmetI *lines for which CpG methylation is altered. As we are currently amplifying our mutant lines in order to get homozygous mutants and characterize their phenotypes and DNA methylation levels, it is still too early to speculate on the observed versus predicted effect of the mutations according to SIFT.

## Conclusion

In the 21st century, the need for crop improvement in order to face the growing demand of modern agriculture is increasing, while the social acceptance of so-called genetically modified or transgenic crops remains low. Besides, many plant species of agronomic importance are still unsuitable for *Agrobacterium*-based insertional mutation techniques, including pea. The development of TILLING technology, based on EMS mutagenesis, can contribute to overcoming this deficiency. Furthermore, as EMS generates an allelic series of the targeted genes it becomes possible to investigate the role of essential genes that are otherwise not likely to be recovered in genetic screens based on insertional mutagenesis. We have developed a complete tool that can be used for both forward (EMS saturated mutant collection and the associated phenotypic database) and reverse (high-throughput TILLING platform) genetics in pea, for both basic science or crop improvement. Hence, by opening it to the community, we hope to fulfill the expectations of both crop breeders and scientists who are using pea as their model of study.

## Materials and methods

### EMS treatment

EMS was diluted to the chosen dose in deionized water. Bottles (Schott type) each containing 900 seeds immersed in 450 ml of deionized water-EMS solution were placed on a rotary shaker (50 rpm) overnight (15 h soaking). The EMS solution was then removed and seeds were rinsed extensively 12 times for 30 minutes with gentle shaking.

### Plant growing conditions

Pea (cultivar Caméor) seeds were sown in pots filled with sterile pouzzolane (inert medium, light volcanic grit) at a sowing depth of about 2 cm followed by abundant watering in greenhouse conditions. Plants were then automatically watered with a solution of 3.5:3.1:8.6 N:P:K. The temperature was maintained between 14°C at night and 30°C during daytime, with supplementary lighting to provide a 16 h day.

### Genomic DNA extraction and pooling

Four pea leaf discs (diameter 10 mm) were collected in 96-well plates containing 2 steel beads (4 mm) per well, and tissues were ground using a bead mill. Genomic DNA was isolated using the DNeasy 96 Plant Kit (Qiagen, Hilden, Germany). All genomic DNA was quantified on a 0.8% agarose gel using λ DNA (Invitrogen, Carlsbad, CA, USA) as a concentration reference. DNA samples were diluted tenfold and pooled eightfold in a 96-well format. A population of 4,704 arrayed DNAs from mutagenized individuals is presently available for screening.

### PCR amplification and mutation detection

PCR amplification was based on nested-PCR and universal primers [14]. The first PCR amplification was a standard PCR reaction using target-specific primers and 4 ng of pea genomic DNA. One microliter of the first PCR served as a template for the second nested PCR amplification, using a mix of gene-specific inner primers carrying a universal M13 tail (CACGACGTTGTAAAACGAC for forward primers; GGATAACAATTTCACACAGG for reverse primers), in combination with M13 universal primers, M13F700 (CACGACGTTGTAAAACGAC) and M13R800 (GGATAACAATTTCACACAGG), labeled at the 5'end with infra-red dyes IRD700 and IRD800 (LI-COR^®^, Lincoln, NE, USA), respectively. This PCR was carried out using 0.1 μM of each primer, using the following two step cycling program: 94°C for 2 minutes, 10 cycles at 94°C for 15 s, primer-specific annealing temperature for 30 s and 72°C for 1 minute, followed by 25 cycles at 94°C for 15 s, 50°C for 30 s and 72°C for 1 minute, then a final extension of 5 minutes at 72°C. Mutation detection was carried out as described previously [28]. The nature of the mutations was identified by sequencing.

## Abbreviations

CODDLE, Codons Optimized to Discover Deleterious Lesions; EMS, ethylmethane sulfonate; PARSESNP, Project Aligned Related Sequences and Evaluate SNPs; SIFT, Sorting Intolerant From Tolerant; TILLING, targeting induced local lesions in genomes.

## Authors' contributions

CLS, FM, JB, GA and RT performed the EMS mutagenesis and took care of the plants; MD extracted the DNA; TILLING screens and analysis were done by MD and JS; VS, VB, YDO and CG set up UTILLdb; AB coordinated the study. The manuscript was written by JS, MD, CLS, VB and AB.

## Additional data files

The following additional data are available with the online version of this paper. Additional data file 1 is a table providing the pea mutant phenotype list used for describing and recording M2 mutant plant phenotypes in UTILLdb.

## Supplementary Material

Additional data file 1 Pea mutant phenotype list used for describing and recording M2 mutant plant phenotypes in UTILLdb. Click here for file

## References

[B1] Hilson P, Allemeersch J, Altmann T, Aubourg S, Avon A, Beynon J, Bhalerao RP, Bitton F, Caboche M, Cannoot B, Chardakov V, Cognet-Holliger C, Colot V, Crowe M, Darimont C, Durinck S, Eickhoff H, de Longevialle AF, Farmer EE, Grant M, Kuiper MTR, Lehrach H, Leon C, Leyva A, Lundeberg J, Lurin C, Moreau Y, Nietfeld W, Paz-Ares J, Reymond P (2004). Versatile gene-specific sequence tags for *Arabidopsis *functional genomics: transcript profiling and reverse genetics applications.. Genome Res.

[B2] Waterhouse PM, Graham MW, Wang M-B (1998). Virus resistance and gene silencing in plants can be induced by simultaneous expression of sense and antisense RNA.. Proc Natl Acad Sci USA.

[B3] Long D, Coupland G (1998). Transposon tagging with Ac/Ds in *Arabidopsis*.. Methods Mol Biol.

[B4] May BP, Liu H, Vollbrecht E, Senior L, Rabinowicz PD, Roh D, Pan X, Stein L, Freeling M, Alexander D, Martienssen R (2003). Maize-targeted mutagenesis: a knockout resource for maize.. Proc Natl Acad Sci USA.

[B5] Alonso JM, Stepanova AN, Leisse TJ, Kim CJ, Chen H, Shinn P, Stevenson DK, Zimmerman J, Barajas P, Cheuk R, Gadrinab C, Heller C, Jeske A, Koesema E, Meyers CC, Parker H, Prednis L, Ansari Y, Choy N, Deen H, Geralt M, Hazari N, Hom E, Karnes M, Mulholland C, Ndubaku R, Schmidt I, Guzman P, Aguilar-Henonin L, Schmid M (2003). Genome-wide insertional mutagenesis of *Arabidopsis thaliana*.. Science.

[B6] Greene EA, Codomo CA, Taylor NE, Henikoff JG, Till BJ, Reynolds SH, Enns LC, Burtner C, Johnson JE, Odden AR, Comai L, Henikoff S (2003). Spectrum of chemically induced mutations from a large-scale reverse-genetic screen in *Arabidopsis*.. Genetics.

[B7] Henikoff S, Till BJ, Comai L (2004). TILLING. Traditional mutagenesis meets functional genomics.. Plant Physiol.

[B8] Comai L, Henikoff S (2006). TILLING: practical single-nucleotide mutation discovery.. Plant J.

[B9] McCallum CM, Comai L, Greene EA, Henikoff S (2000). Targeting induced local lesions in genomes (TILLING) for plant functional genomics.. Plant Physiol.

[B10] Bentley A, MacLennan B, Calvo J, Dearolf CR (2000). Targeted recovery of mutations in *Drosophila*.. Genetics.

[B11] Coghill EL, Hugill A, Parkinson N, Davison C, Glenister P, Clements S, Hunter J, Cox RD, Brown SDM (2002). A gene-driven approach to the identification of ENU mutants in the mouse.. Nat Genet.

[B12] Colbert T, Till BJ, Tompa R, Reynolds S, Steine MN, Yeung AT, McCallum CM, Comai L, Henikoff S (2001). High-throughput screening for induced point mutations.. Plant Physiol.

[B13] Perry JA, Wang TL, Welham TJ, Gardner S, Pike JM, Yoshida S, Parniske M (2003). A TILLING reverse genetics tool and a web-accessible collection of mutants of the legume *Lotus japonicus*.. Plant Physiol.

[B14] Wienholds E, van Eeden F, Kosters M, Mudde J, Plasterk RHA, Cuppen E (2003). Efficient target-selected mutagenesis in zebrafish.. Genome Res.

[B15] Menda N, Semel Y, Peled D, Eshed Y, Zamir D (2004). *In silico *screening of a saturated mutation library of tomato.. Plant J.

[B16] Miyao A, Iwasaki Y, Kitano H, Itoh J-I, Maekawa M, Murata K, Yatou O, Nagato Y, Hirochika H (2007). A large-scale collection of phenotypic data describing an insertional mutant population to facilitate functional analysis of rice genes.. Plant Mol Biol.

[B17] Kuromori T, Wada T, Kamiya A, Yuguchi M, Yokouchi T, Imura Y, Takabe H, Sakurai T, Akiyama K, Hirayama T, Okada K, Shinozaki K (2006). A trial of phenome analysis using 4000 Ds-insertional mutants in gene-coding regions of *Arabidopsis*.. Plant J.

[B18] Lawrence CJ, Seigfried TE, Brendel V (2005). The Maize Genetics and Genomics Database. The community resource for access to diverse maize data.. Plant Physiol.

[B19] Lee JM, Davenport GF, Marshall D, Ellis THN, Ambrose MJ, Dicks J, van Hintum TJL, Flavell AJ (2005). GERMINATE. A generic database for integrating genotypic and phenotypic information for plant genetic resource collections.. Plant Physiol.

[B20] Rhee SY, Beavis W, Berardini TZ, Chen G, Dixon D, Doyle A, Garcia-Hernandez M, Huala E, Lander G, Montoya M, Miller N, Mueller LA, Mundodi S, Reiser L, Tacklind J, Weems DC, Wu Y, Xu I, Yoo D, Yoon J, Zhang P (2003). The *Arabidopsis *Information Resource (TAIR): a model organism database providing a centralized, curated gateway to *Arabidopsis *biology, research materials and community.. Nucleic Acids Res.

[B21] Domoney C, Duc G, Ellis TN, Ferrandiz C, Firnhaber C, Gallardo K, Hofer J, Kopka J, Kuster H, Madueno F, Munier-Jolain NG, Mayer K, Thompson R, Udvardi M, Salon C (2006). Genetic and genomic analysis of legume flowers and seeds.. Curr Opin Plant Biol.

[B22] The Grain Legumes Integrated Project. http://www.eugrainlegumes.org/.

[B23] Loridon K, McPhee K, Morin J, Dubreuil P, Pilet-Nayel M, Aubert G, Rameau C, Baranger A, Coyne C, Lejeune-Hénaut I, Burstin J (2005). Microsatellite marker polymorphism and mapping in pea (*Pisum sativum *L.).. Theor Appl Genet.

[B24] Aubert G, Morin J, Jacquin F, Loridon K, Quillet M, Petit A, Rameau C, Lejeune-Hénaut I, Huguet T, Burstin J (2006). Functional mapping in pea, as an aid to the candidate gene selection and for investigating synteny with the model legume *Medicago truncatula*.. Theor Appl Genet.

[B25] Till BJ, Reynolds SH, Greene EA, Codomo CA, Enns LC, Johnson JE, Burtner C, Odden AR, Young K, Taylor NE, Henikoff JG, Comai L, Henikoff S (2003). Large-scale discovery of induced point mutations with high-throughput TILLING.. Genome Res.

[B26] CODDLE: Codons Optimized to Discover Deleterious LEsions. http://www.proweb.org/coddle.

[B27] Rozen S, Skaletsky H, Krawetz SA, Misener S (2000). Primer3 on the WWW for general users and for biologist programmers.. Bioinformatics Methods and Protocols: Methods in Molecular Biology.

[B28] Triques K, Sturbois B, Gallais S, Dalmais M, Chauvin S, Clepet C, Aubourg S, Rameau C, Caboche M, Bendahmane A (2007). Characterization of *Arabidopsis thaliana *mismatch specific endonucleases: application to mutation discovery by TILLING in pea.. Plant J.

[B29] Kankel MW, Ramsey DE, Stokes TL, Flowers SK, Haag JR, Jeddeloh JA, Riddle NC, Verbsky ML, Richards EJ (2003). *Arabidopsis *MET1 cytosine methyltransferase mutants.. Genetics.

[B30] Krieg DR (1963). Ethyl methanesulfonate-induced reversion of bacteriophage T4rII mutants.. Genetics.

[B31] Markiewicz P, Kleina LG, Cruz C, Ehret S, Miller JH (1994). Genetic studies of the lac repressor. XIV. Analysis of 4000 altered *Escherichia coli *lac repressors reveals essential and non-essential residues, as well as "spacers" which do not require a specific sequence.. J Mol Biol.

[B32] Altschul S, Gish W, Miller W, Myers E, Lipman DJ (1990). Basic local alignment search tool.. J Mol Biol.

[B33] UTILLdb: URGV TILLING database. http://urgv.evry.inra.fr/UTILLdb.

[B34] Ilic K, Kellogg EA, Jaiswal P, Zapata F, Stevens PF, Vincent LP, Avraham S, Reiser L, Pujar A, Sachs MM, Whitman NT, McCouch SR, Schaeffer ML, Ware DH, Stein LD, Rhee SY (2007). The Plant Structure Ontology, a unified vocabulary of anatomy and morphology of a flowering plant.. Plant Physiol.

[B35] Jaiswal P, Avraham S, Ilic K, Kellogg E, McCouch S, Pujar A, Reiser L, Rhee S, Sachs M, Schaeffer M, Stein L, Stevens P, Vincent L, Ware D, Zapata F (2005). Plant Ontology (PO): a controlled vocabulary of plant structures and growth stages.. Comp Funct Genomics.

[B36] Caldwell DG, McCallum N, Shaw P, Muehlbauer GJ, Marshall DF, Waugh R (2004). A structured mutant population for forward and reverse genetics in Barley (*Hordeum vulgare *L.).. Plant J.

[B37] Nakamura Y, Gojobori T, Ikemura T (2000). Codon usage tabulated from international DNA sequence databases: status for the year 2000.. Nucleic Acids Res.

[B38] Gilchrist E, O'Neil N, Rose A, Zetka M, Haughn G (2006). TILLING is an effective reverse genetics technique for *Caenorhabditis elegans*.. BMC Genomics.

[B39] Till B, Cooper J, Tai T, Colowit P, Greene E, Henikoff S, Comai L (2007). Discovery of chemically induced mutations in rice by TILLING.. BMC Plant Biol.

[B40] Till B, Reynolds S, Weil C, Springer N, Burtner C, Young K, Bowers E, Codomo C, Enns L, Odden A, Greene E, Comai L, Henikoff S (2004). Discovery of induced point mutations in maize genes by TILLING.. BMC Plant Biol.

[B41] Slade AJ, Fuerstenberg SI, Loeffler D, Steine MN, Facciotti D (2005). A reverse genetic, nontransgenic approach to wheat crop improvement by TILLING.. Nat Biotechnol.

[B42] Ng PC, Henikoff S (2003). SIFT: predicting amino acid changes that affect protein function.. Nucleic Acids Res.

[B43] Taylor NE, Greene EA (2003). PARSESNP: a tool for the analysis of nucleotide polymorphisms.. Nucleic Acids Res.

[B44] Xiao W, Custard KD, Brown RC, Lemmon BE, Harada JJ, Goldberg RB, Fischer RL (2006). DNA methylation is critical for *Arabidopsis *embryogenesis and seed viability.. Plant Cell.

[B45] Ellis THN, Poyser SJ (2002). An integrated and comparative view of pea genetic and cytogenetic maps.. New Phytologist.

[B46] Martienssen RA, Colot V (2001). DNA methylation and epigenetic inheritance in plants and filamentous fungi.. Science.

