# Supplementary material for: UTILLdb, a Pisum sativum in silico forward and reverse genetics tool
Source: Genome Biol. 2008 Feb 26;9(2):R43. doi: 10.1186/gb-2008-9-2-r43 (PMC2374714; doi:10.1186/gb-2008-9-2-r43)
Supplement: Additional data file 1 — Pea mutant phenotype list used for describing and recording M2 mutant plant phenotypes in UTILLdb. [file gb-2008-9-2-r43-S1.pdf]

Table 1-suppl: Pea mutants phenotypic list

	Major categ	Sub catego	Sub sub caregory	no.of families
1	Cotyledon	Color	yellow variegated	48
			yellow bright	25
			albino	53
			dark	5
			green variegated	41
		Shape	curling	26
			waxy	6
2	Plantlet arc	Architectur	Bushy	5
			Giant	2
3	Plant archi	Architectur	compact	120
			giant with normal inter	22
			miniature	119
			long internodes	1
			giant with long internoc	3
			compact at the top	1
			hyper compact	50
		Branching	basal branching	94
			determinate growth	14
			branching	85
			bushy	12
4	Leaf	Color	vein/lam - veining	20
			glossy	76
			dark green	38
			variegated	88
			pale green	325
			silver-argenteum	45
			yellow	18
		Shape and	upcurling	108
			indented edge	24
			round	25
			downcurling	33
			afila - tendrils only	2
			coneshaped at leaf ba	20
			leaf ended by tendrils	17
			margin raised	8
			no tendril	1
			leaflets superimposed	7
			rhomboid-pointed	34
			distorted	64
			long tendrils	1
			multifoliate	43
		Appearanc	wilted	10
			spotted	1
			thick	11
			waxy	2
			crinkled	53
			stunted	144
			mottled	32
		Size	narrow	39
			medium	36
			very narrow	6
5	Stipule	Size/Color/	indented	30

			pointed	3
			narrow	10
			round	2
			asymmetric	3
			large	4
			distorted	14
			silver-argenteum	2
			white variegated	9
6	Petiole	Petiole	short	4
			absence	2
7	Stem	Stem size	extrem dwarf	422
			dwarf(1/4-2/3 of n)	1025
		Shape	thin	32
			fasciated	4
8	Flowers	Flower mor	abnormal petals	3
			abnormal all	18
			cauliflower type inflore	3
		Flowering t	early flowering	4
		Reproducti	sterile flowers	10
			no flower	2
9	Seed	Seed color	beige	1
			pale	1
		Shape	wrinkled	1
			crackled seed coat	1
			extremely wrinkled	2
		Size	large	17
			small	49
